# A Gemini Virus-Derived Autonomously Replicating System for HDR-Mediated Genome Editing of the *EPSP Synthase* Gene in Indica Rice

**DOI:** 10.3390/plants14030477

**Published:** 2025-02-06

**Authors:** Bhabesh Borphukan, Muslima Khatun, Dhirendra Fartyal, Donald James, Malireddy K. Reddy

**Affiliations:** 1 Department of Crop and Soil Sciences, Washington State University, Pullman, WA 99163, USA; 2 Crop Improvement Group, International Centre for Genetic Engineering and Biotechnology, New Delhi 110067, India; reddy@icgeb.res.in; 3 Molecular Biotechnology Division, National Institute of Biotechnology, Dhaka 1349, Bangladesh; muslima@nib.gov.bd; 4 Plant Nutrition Division, INRES, University of Bonn, 53113 Bonn, Germany; dhirenfartyal@gmail.com; 5 Department of Biotechnology, Forest Genetics and Biotechnology Division, Kerala Forest Research Institute, Thrissur 680653, India; donjamdj23@gmail.com

**Keywords:** genome editing, CRISPR/Cas9, homology directed repair, viral replicon, wheat dwarf virus, *OsEPSPS* gene

## Abstract

CRISPR/Cas9-mediated homology-directed repair (HDR) is a powerful tool for precise genome editing in plants, but its efficiency remains low, particularly for targeted amino acid substitutions or gene knock-ins. Successful HDR requires the simultaneous presence of Cas9, guide RNA, and a repair template (RT) in the same cell nucleus. Among these, the timely availability of the RT at the double-strand break (DSB) site is a critical bottleneck. To address this, we developed a sequential transformation strategy incorporating a deconstructed wheat dwarf virus (dWDV)-based autonomously replicating delivery system, effectively simplifying the process into a two-component system. Using this approach, we successfully achieved the targeted editing of the *OsEPSPS* gene in rice with a 10 percent HDR efficiency, generating three lines (TIPS1, TIPS2, and TIPS3) with amino acid substitutions (T172I and P177S) in the native EPSPS protein. The modifications were confirmed through Sanger sequencing and restriction digestion assays, and the edited lines showed no yield penalties compared to wild-type plants. This study demonstrates the utility of viral replicons in delivering gene-editing tools for precise genome modification, offering a promising approach for efficient HDR in crop improvement programs.

## 1. Introduction

The success of conventional plant breeding relies heavily on the presence of genetic variations within the population of a crop species to facilitate the introgression of the beneficial traits into an elite cultivar [1]. However, transferring beneficial alleles from genetically diverse backgrounds, such as landraces or wild relatives, into commercial cultivars is time-consuming and often results in linkage drag, introducing unwanted alleles alongside beneficial ones [2]. Although the introduction of foreign DNA into plant genomes through a transgenic approach has been a focus of crop improvement [3], the transgenic approach does not harness a plant’s native genetic repertoire to create traits of agricultural value. Therefore, public concerns over the incorporation of transgene from distantly related organisms into crop plants and the governments’ regulatory safety concerns have impeded the widespread use of the transgenic approach for crop improvement [4].

With the advanced demonstration of the CRISPR/Cas9 system as a tool for crop genome modification, it has become easier to introduce the required genetic variability into any established cultivar directly by targeted genome editing without any requirement of actual mutant lines and the time-consuming plant breeding processes [5]. The Cas9 introduces double-strand DNA breaks (DSBs) in any defined region of the genome, guided by 20 base gRNA sequences [6,7,8]. These DNA breaks are frequently repaired by the DNA damage repair machinery existing in all eukaryotic systems. The double-stranded DNA breaks are most frequently repaired through non-homologous end-joining (NHEJ) processes, and this process often results in the deletion or addition of a few nucleotides, causing a frameshift and/or gene knockout [9,10]. Alternatively, DSBs can be repaired by the homology-directed repair (HDR) mechanism, where a repair template with homologous sequences facilitates precise sequence modification or gene knock-in [11,12]. However, the precise selective substitution of nucleotide sequences using HDR-mediated genome editing is more challenging to implement because the cleavage of DNA by Cas9 must be coordinated with the delivery of a DNA repair template and subsequent selection and/or identification of properly edited cell line or plant [13,14].

NHEJ is the most common double-strand break repair mechanism in most organisms, including higher plants [15,16]. Previously, some success has been reported in enhancing HDR efficiency by suppressing the NHEJ pathway both in vitro and in vivo [17,18,19,20]. The delivery of a stable and persistent repair template or donor DNA remains a significant hurdle in achieving efficient HDR in higher plants [21,22,23]. Moreover, the delivery of genome editing components through *Agrobacterium*-mediated transformation into plant tissue is not very efficient because the transformed repair template, as a part of the T-DNA region, often integrates into the plant genome, making it unavailable for HDR-mediated DNA repair. The screening and identification of successfully knock-in edited cell lines and the subsequent regeneration of a viable plant is another difficulty. These factors make it difficult to successfully utilize HDR-mediated genome editing in plant systems. Despite these difficulties, HDR-mediated genome editing has the higher potential to introduce precise, user-defined genetic variabilities, facilitating targeted trait manipulation in crop plants.

To address these critical rate-limiting factors associated with gene knock-in in plants, we developed an autonomously replicating wheat dwarf virus (WDV)-derived delivery system to enhance HDR efficiency for precision crop improvement. We targeted the rice gene encoding for EPSP synthase for selective amino acid substitution to create a rice plant resistant to the broad-spectrum systemic herbicide glyphosate. Glyphosate is a strong inhibitor of EPSP synthase, a critical plant enzyme involved in the biosynthesis of aromatic amino acids. The availability of sound knowledge about the mode of molecular inhibitory interaction of this herbicide, and the availability of genetic and molecular information regarding the herbicide-resistant mechanism suggests the desired sequence modifications to edit for herbicide tolerance in any given crop plant. The double amino acid substitution (T173I and P177S) mutant EPSP synthase confers significant resistance to glyphosate [24]. These amino acid substitutions in rice EPSP synthase are expected to reduce or destroy the ability of the herbicide glyphosate to interact with the (T173I and P177S) mutant EPSP synthase, while simultaneously retaining the capacity for normal functioning in the presence of herbicide.

## 2. Results

Here, we aimed to introduce two specific amino acid substitutions (T173I and P177S) within the native rice *EPSP synthase* gene using a deconstructed virus genome-based autonomous replication system. This approach was intended to reduce reliance on traditional *Agrobacterium*-mediated T-DNA delivery for gene knock-in, enabling more efficient and stable genetic modifications to enhance key agronomic traits in rice.

### 2.1. Development of Stable rcoSpCas9 Overexpression Rice Lines

**Figure 1:**
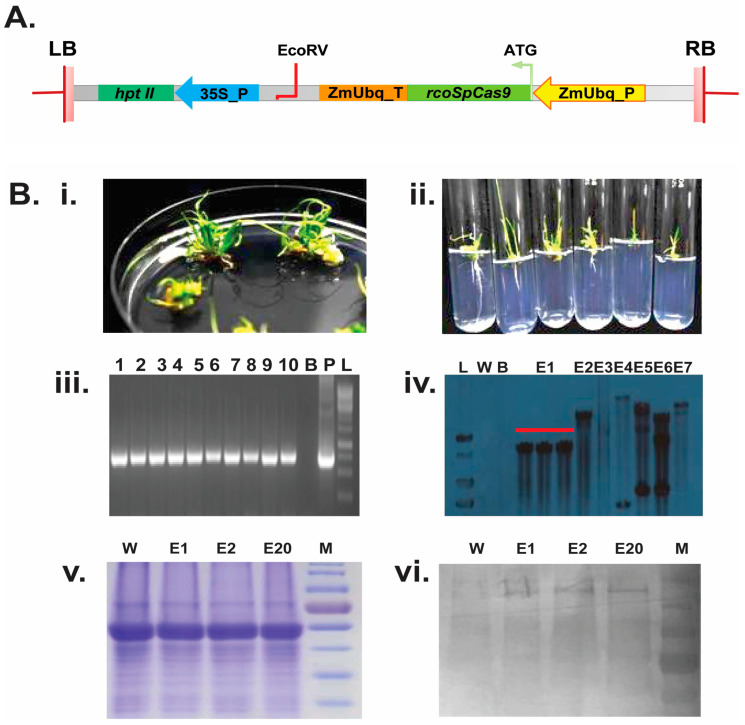
(**A**) rcoSpCas9 expression cassette and first round of plant transformation: LB/RB: T-DNA left and right border; hptII- *Hygromycin* coding sequence (CDS); 35S_P: Promoter of Cauliflower mosaic virus 35S gene; ZmUbq_T- Maize ubiquitin gene terminator, ZmUbq_P- *Zea maiz Ubiquitin* gene promoter rcoSpCas9- Rice codon-optimized *Streptococcus pyrogens Cas9* CDS. (**B**) Development of stable lines and molecular confirmation of rcoSpCas9 expressing MTU1010 transgenic rice (i) and (ii): Regeneration and rooting of putative transgenic lines; (iii): 1–10 putative rcoSpCas9 transgenic lines confirmed through PCR; B: blank lane; P: pMDC99:rcoSpCas9 plasmid as positive control; L: 1 kb DNA ladder (GeneRuler) (iv): Southern blot analysis of PCR positive control lines; L: 1 kb DNA ladder (GeneRuler); W: wild type MTU1010 DNA as negative control; B: blank lane; E1–E20: PCR positive rcoSpCas9 lines (v): Coomassie-stained SDS-PAGE gel showing equal loading of total protein extracted from E1, E2, E20 and W lines. The gel serves as a loading control to confirm uniform protein quantities across lanes. (vi) Western blot analysis of the same gel, probed with an anti-Cas9 antibody, confirming the expression of rcoSpCas9 protein in the transgenic lines (E1, E2, and E20). The position of the Cas9 protein (~expected 160 kDa) and W: wild type MTU1010 total soluble protein as negative control, E1, E2, and E20: Southern positive rcoSpCas9 expressed lines; M- Protein ladder.

In this study, we targeted the *EPSP synthase* gene in rice (*Oryza sativa* spp. *Indica* cv MTU1010) for selective amino acid substitutions (T173I and P177S or together TIPS) using the CRISPR/Cas9 system. To achieve this, we first generated stable rice lines overexpressing rice codon-optimized *Streptococcus pyrogens* Cas9 (rcoSpCas9) under the control of a constitutive *Zea maize* Ubiquitin promoter (ZmUbq_P) (Figure 1A,B). Initially, three-week-old rice calli were transformed with the rcoSpCas9 overexpression cassette, resulting in 20 putative transgenic lines (pMDC99:rcoSpCas9: NosT). These newly generated transgenic lines (T0) were further confirmed through PCR with the primer pairs Zm JN_F/Cas9 JN_R (Table S1) targeting the maize ubiquitin promoter and rcoSpCas9 (Figure 1B, iii) as well as the hygromycin region (hptII F/hptII R, Table S1). T0-Seeds were germinated under 50 mg/L hygromycin selection (Figure S3) and subsequently validated through southern blot and western blot analysis (Figure 1B, iv and vi). Based on these analyses, we selected the E1, E2, and E20 stably-overexpressed rcoSpCas9 lines for the second round of transformation.

### 2.2. Development of the dWDV Replicon-Based Repair Template Delivery System

**Figure 2:**
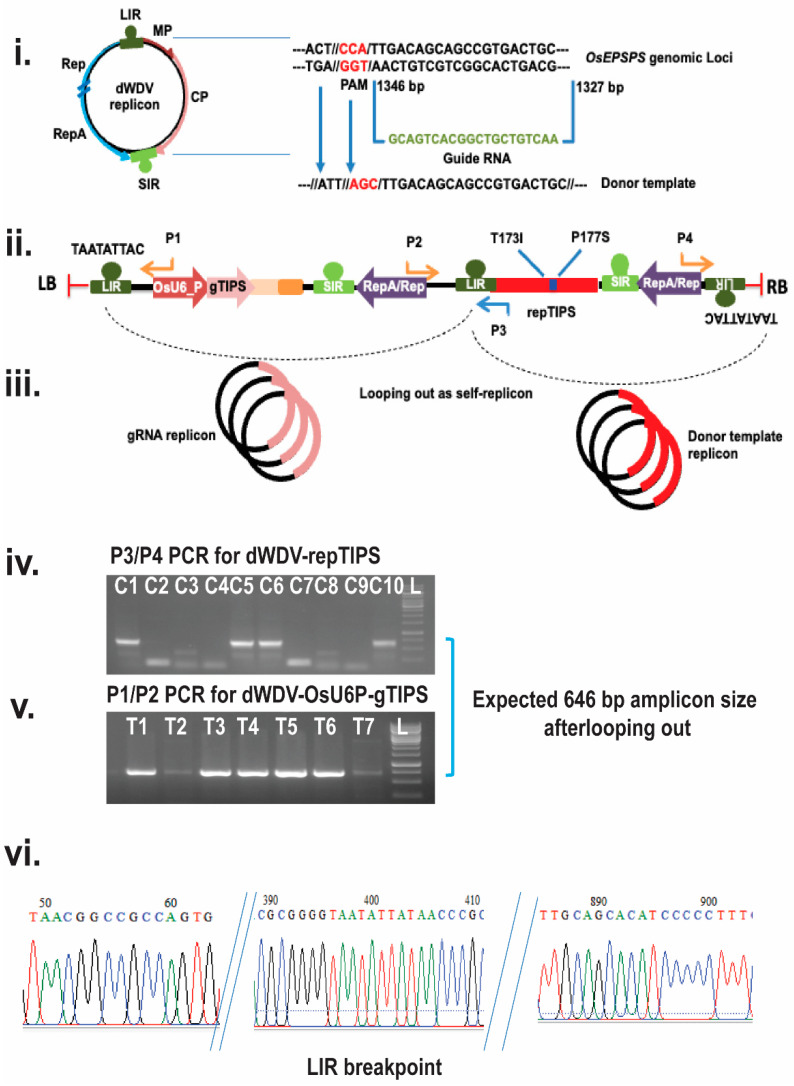
Designing of deconstructed wheat dwarf virus (dWDV)-based autonomously replicated delivery system and confirmation of looping out (**i**). Orientation WDV genome and genomic loci of rice EPSPS gene target region with guide RNAs (gTIPS) and homology-directed repair/donor (HDR) template (**ii**). Expression cassettes of pMDC99:dWDV-OsU6_P:gTIPS::dWDV-repTIPS; LIR: long intergenic region, gTIPS- guide RNA targeting OsEPSPS gene; OsU6_P: Rice RNA pol III U6 promoter; OsPol III_T: Rice RNA polymerase III terminator, SIR: Short intergenic region, RepA/Rep: Replication associated protein, repTIPS: donor template carrying TIPS substitution (T173I and P177S mutation); LB: Left border of T-DNA and RB: Right borer of T-DNA (**iii**). Graphical abstract of looping out of guide RNA and donor template in rice calli (**iv**) and (**v**). PCR confirmation of looping out from DNA collected from second round of rice calli transformation; C1–C10: PCR amplicone from P3/P4 primer pair (~646 bp PCR product from dWDV-repTIPS replicon); T1–T7: PCR amplicone from P1/P2 primer pair (~646 bp PCR product from dWDV-OsU6P-gTIPS replicon) (**vi**). further sequenced confirmation of proper looping out with LIR breakpoint.

A wheat dwarf virus (WDV)-derived replicon system was developed to provide a persistent repair template for HDR. First, the WDV genome was linearized at the potential stem–loop region (TAATATTAT) of the long intergenic region (LIR) and, subsequently, the coat protein-encoding region was replaced with a selected rice *EPSP synthase* donor template (repTIPS/706 bp) harboring selective T173I/P177S substitutions. Essential viral replication components, such as the short intergenic region (SIR) and replication protein (Rep/RepA) (Figure 2, iii), were retained to maintain autonomous replication. The deconstructed WDV (dWDV)-based donor template delivery system (dWDV:repTIPS) was synthesized with all the necessary components and cloned into our lab-modified multi-gateway compatible entry vector (pL12R34-Ap) under *Eco*RI and *Sac*I restriction sites. Similarly, a gRNA expression cassette under the rice U6 promoter and the pol-III terminator (dWDV: OsU6_P:gTIPS) was cloned into an entry vector (pL34R12-Cm-ccdB). Finally, the multi-round gateway cloning method was used to stack the components into the final plant transformation vector pMDC99:: dWDV:repTIPS:: dWDV:OsU6_P:gTIPS for transformation into a *rcoSpCas9*-expressing callus (Figure 2, ii and iii).

### 2.3. Verification of Autonomous Replication of the dWDV Replicon

The existence of the desired circular copies of the viral genome carrying the repair template is a foremost requirement for HDR-mediated knock-in at the targeted double-strand DNA break created by the Cas9-gTIPS duplex. To check for the looping out of the dWDV-based gRNA and donor template delivery system into an autonomous circular replicon (Figure 2, ii and iii), we isolated total nucleic acids from the calli generated from the second round of transformation. Rice calli selected for total DNA isolation were harvested after the third round of selection. To check the autonomous replicating body within the calli, we designed an oppositely oriented PCR primer pair targeting regions within both the guide RNA and repair template separately (primer details in Table S1). The PCR amplification of a unique 646 bp DNA fragment was obtained (Figure 2, iv and v), as expected, from the oppositely oriented primer pair (P1/P2 and P3/P4). This PCR amplification is possible only when the dWDV replicon is looping out from the T-DNA integration site and available as a circular DNA in the rice calli (Figure 2, ii). This proved our hypothesis of the generation of an autonomous replication system. Further, we cloned the 646 bp amplicon into the pCRtopo2.1 vector (Invitrogen), and Sanger sequenced both strands for validation. The sequencing analysis (Figure 2, vi) confirmed the expected sequence information, including an intact LIR breakpoint.

### 2.4. Generation and Validation of TIPS-Edited Plants

**Figure 3:**
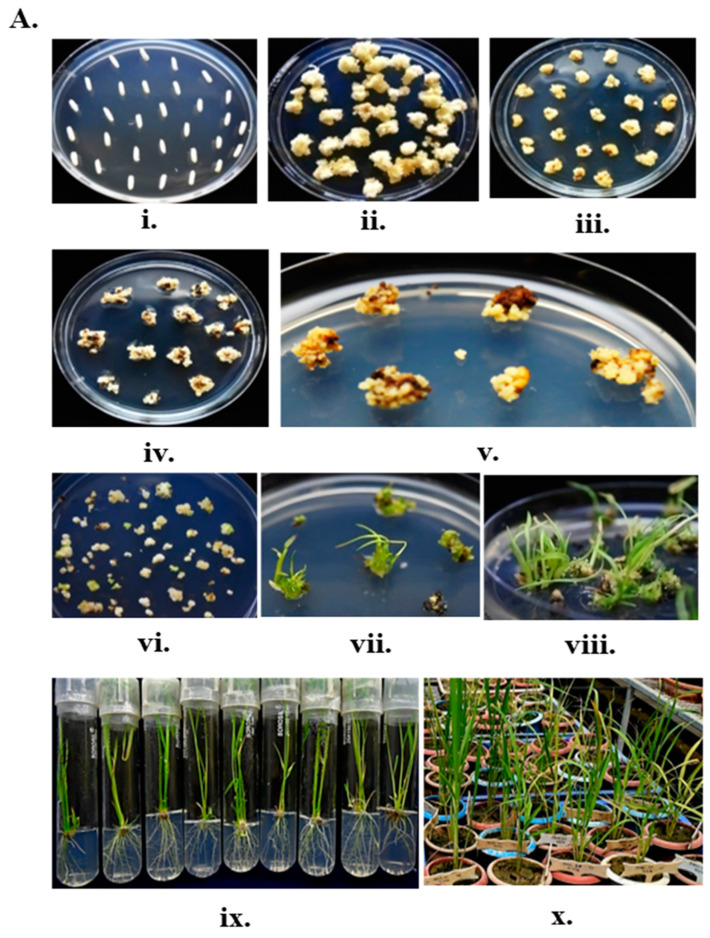

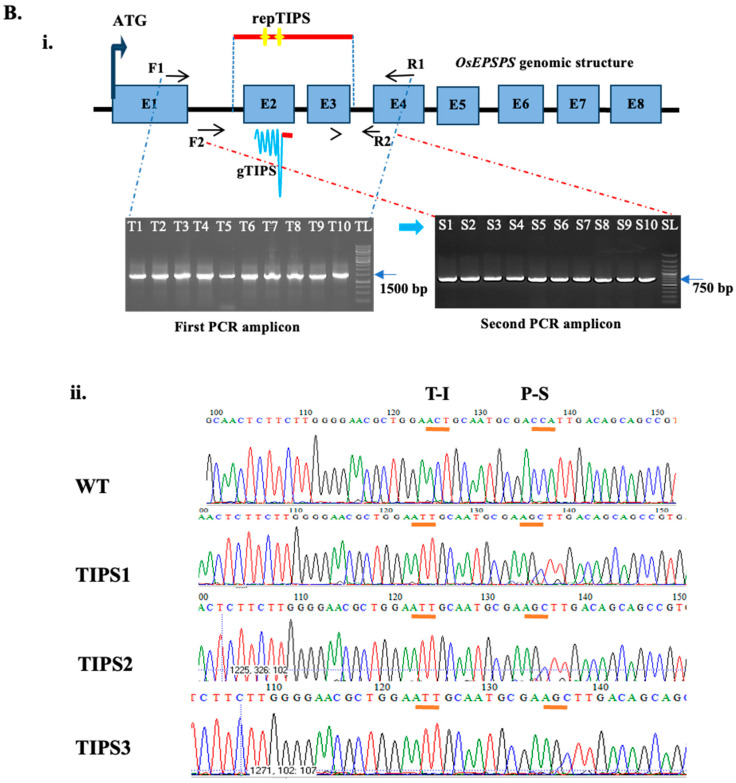
(**A**) Second round transformation under glyphosate selection using pMDC99:: dWDV:repTIPS:: dWDV:OsU6_P:gTIPS construct.: (i–iii). Callus induction under 35 mg/L hygromycin (iv–vi). First to third selection under 50 µmol/L glyphosate selection (vii–x). Regeneration under 25 µmol/L glyphosate selection. (**B**) Target amplification and mutation confirmation through sequencing and target site digestion (i). T1–T10—F1/R1 primer (first) PCR products, TL—GeneRuler 1kb DNA ladder; S1–S10—F2/R2 primer (second) PCR products, SL—GeneRuler 100 bp DNA ladder (ii). Sequencing confirmation of WT—MTU1010 control plant, TIPS1/2/3—OsEPSPS edited lines for T173I and P177S substitution.

Following the second round of transformation using the seeds collected from the stably overexpressed rcoSpCas9 lines E1, E2, and E20, new plants were regenerated from calli selected on MS media containing glyphosate (25 µmol/L) (Figure 3A). We successfully generated 30 putative edited lines. These putative rice lines were analyzed for precise substitution of (T173I and P177S) amino acids in the native *EPSP synthase* encoding gene by Sanger sequencing.

The target sequence was PCR amplified using a nested PCR strategy with two specific primer pairs (OsEPSPS mut_F1/mut_R1 and OsEPSPS mut_F2/mut_R2; Table S1) flanking the designated target region of the rice EPSP synthase encoding gene. The first PCR primer set (OsEPSPS_mut F1/R1, ~1500 bp) was annealed specifically to the rice *EPSP synthase* native copy, strategically designed to avoid amplifying the donor template introduced through the dWDV replicon (dWDV:repTIPS) (Figure 3B, i). Sanger sequencing was performed using the PCR amplicon of the second set of primer (EPSPS_mut F2/R2, ~500 bp). After sequence analysis, we found three individual rice plants (designated hereafter as TIPS1, TIPS2, and TIPS3) out of 30 green seedlings obtained in this study that showed precise T173I and P177S substitution in the *OsEPSPS* gene, suggesting successful homology-directed repair through the user-defined repair template (Figure 3B, ii).

**Figure 4:**
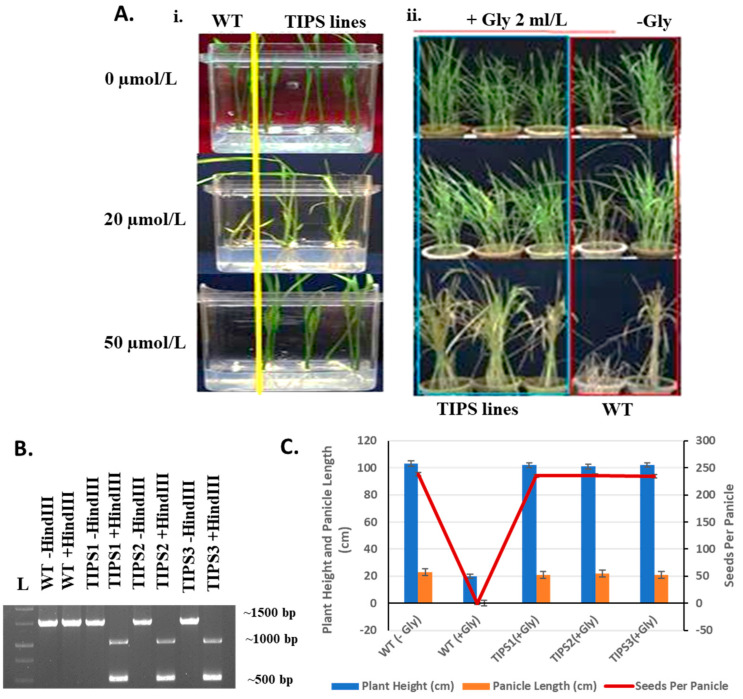
(**A**) i. Mutation stability and inheritance analysis of edited TIPS mutant lines. (i). T1 seeds from edited lines TIPS 1/2/3 were grown in MS media containing glyphosate in 0, 20, and 50 micromole concentrations. WT: MTU1010 control seeds; TIPS: TIPS 1, 2, and 3 lines (ii). Pot experiment to test Roundup (commercial glyphosate) aerial spray. A total of 2 mL per liter concentration of commercial Roundup was sprayed on the aerial part at the early tillering stage. WT: MTU1010 control plants, +Gly: Roundup application; −Gly: Mock spray. (**B**) Restriction digestion of target site amplicon with *Hind*III enzyme for mutation screening. L: 1 kb DNA ladder; WT− *Hind*III and WT+ *Hind*III: Target site amplicon from control plant MTU1010 without *Hind*III digestion and with digestion, TIPS1/2/3—*Hind*III/+ *Hind*III: Target site amplicon from TIPS mutant lines without and with *Hind*III digestion. (**C**) Graphical representation of statistical analysis of plant height (PH), panicle length (PL), and seed per panicle of WT: MTU1010 control plant and TIPS-1/2/3 mutant lines with (+Gly, 2 mL/L Roundup) and without (−Gly, mock spray).

This result demonstrated successful gene knock-in events in the MTU1010 rice background at the relatively high rate of 10 percent HDR efficiency. Additionally, our PCR strategy proved highly effective in eliminating any amplicon derived from the delivered engineered repair template present under the T-DNA border or the autonomously replicating component. The introduced unique *Hin*dIII restriction site alongside the TIPS mutation in the donor template also served as an additional validation marker for the screening of successive edited lines (Figure 4B). 

### 2.5. Assessment of Glyphosate Tolerance and Agronomic Traits

To evaluate glyphosate tolerance and assess agronomic traits, the T1 seeds from TIPS-edited lines and wild-type (WT) MTU1010 were germinated on MS media with varying concentrations of glyphosate (0, 20, and 50 µmol/L). Wild-type (WT) rice seedlings exhibited significant growth inhibition after the initial germination at 50 µmol/L glyphosate concentration, whereas the TIPS-edited lines (TIPS 1/2/3) displayed normal growth (Figure 4A, i). In addition to the germination test, we further assessed the performance of TIPS-edited lines against the commercially available glyphosate herbicide, Roundup. All the confirmed edited rice lines and control, WT lines (MTU1010) were grown in separate earthen pots in the greenhouse transplanting seedlings after 25 days of germination. At the early tillering stage (~20 days after transplantation), we sprayed the recommended concentration (2 mL/L) of the herbicide Roundup (Figure 4A, ii). The TIPS-edited rice lines showed normal growth without any crop injury after spraying and subsequently matured and set seeds without any yield penalty (Figure 4C). However, apparent herbicide injury symptoms were visible 7 days after the herbicide Roundup was sprayed on the control WT (MTU1010) lines. Subsequently, the WT lines died completely within the next 10–12 days of herbicide application (Figure 4C). Analysis of important parameters, i.e., plant height (cm), panicle length (cm), and seeds per panicle showed no significant difference in the performance of TIPS-edited lines under glyphosate treatment when compared to mock-treated WT plants (Figure 4C).

## 3. Discussion

In this study, we achieved targeted amino acid substitutions (T173I and P177S, or TIPS) within the native *EPSP synthase* gene in rice (*Oryza sativa ssp. Indica* cv MTU1010) using a CRISPR/Cas9-mediated HDR approach. Our findings build upon previous work demonstrating that transgenic overexpression of *OsEPSPS* with TIPS substitutions retains enzymatic function in the presence of glyphosate [24].

In our study, we first generated stable *rcoSpCas9*-expressing rice lines under a strong constitutive *Zea mayze* Ubiquitin promoter (ZmUbq_P), providing a reliable source of Cas9 protein for subsequent editing. Previous studies have shown that constitutive Cas9 expression increases the likelihood of achieving the desired edits in plant systems [25,26,27]. In this work, our Cas9-expressing lines (E1, E2, and E20) were instrumental in enabling HDR-mediated editing when combined with a deconstructed wheat dwarf virus (dWDV) replicon system. Moreover, HDR-mediated editing in plants is often hindered by the dominance of NHEJ repair pathways and the transient availability of repair templates [28]. In our study, we addressed these challenges by using a dWDV-derived replicon system, which provided a stable source of the repair template synchronized with Cas9-induced double-strand breaks (DSBs). Our results align with findings from other studies using Gemini virus replicons to improve HDR efficiency in plant species [27,29,30]. The circular, autonomously replicating dWDV system allowed us to overcome the limitations of *Agrobacterium*-mediated transformation, which often results in stable integration of T-DNA, thereby limiting template availability for HDR [31]. Our PCR amplification of a 646 bp fragment confirmed the formation of a circular, autonomously replicating dWDV replicon in a transformed callus, and sequencing analysis validated its structure, ensuring that the repair template was available in the correct form for efficient HDR.

The successful introduction of T173I and P177S substitutions in the *EPSP synthase* gene of rice provides effective glyphosate tolerance without compromising agronomic performance. Glyphosate is a widely used herbicide that inhibits EPSP synthase in the shikimate pathway, essential for aromatic amino acid synthesis in plants [32,33]. The precise editing achieved in this study allows for herbicide tolerance in rice without foreign DNA integration, aligning with other studies that used endogenous gene editing to confer herbicide resistance in crops [34,35,36,37]. This approach not only enhances crop resilience but also will be useful in addressing regulatory concerns regarding transgene incorporation in crops. One of the advantages of our dWDV-based HDR approach is its potential to create non-transgenic, precisely edited plants. As regulatory frameworks evolve to distinguish between genome-edited and transgenic crops, genome editing without foreign DNA integration could expedite regulatory approval and improve public acceptance [38,39,40]. By avoiding stable T-DNA integration and employing a replicon-based repair template, our approach aligns with recent advancements in Latin America, where countries like Argentina lead in the adoption and field trials of genome-edited crops for research and propagation [41,42].

Moreover, the dWDV replicon system has broader applications for precision breeding, particularly in traits where exact genetic modifications are essential. Gemini virus replicons have shown success in enhancing HDR across a range of crops, including monocots and dicots like rice, wheat, maize, citrus, and grape [28,43,44,45,46]. By enabling targeted editing of endogenous genes without foreign DNA, the replicon system is well-suited for advancing complex trait engineering and precision breeding. Future studies could optimize this system further by integrating multiplexed gRNA constructs for simultaneous edits, making it an adaptable platform for high-efficiency genome editing in agriculture [20,47].

## 4. Materials and Methods

### 4.1. Plant Materials, Transformation and Growth Conditions

Seeds of *Oryza sativa L ssp. Indica* cv. MTU1010 (Cotton Dora Sannalu), an elite, high-yielding variety with long, slender grains that is derived from a cross between Krishnaveni and IR-64 (year of released 2000) (https://drdpat.bih.nic.in), were obtained from the Andhra Pradesh Rice Research Institute (APRRI), Maruteru, India. Seeds of MTU1010 were used for two consecutive transformation events: the first with a rice codon-optimized *Streptococcus pyrogens Cas9* (*rcoSpCas9*) overexpression cassette, and the second with a deconstructed wheat dwarf virus (dWDV) delivery cassette. The transformation with the *rcoSpCas9* overexpression cassette was selected with 50 mg/L hygromycin concentration and calli from the second transformation event were selected using 25 µmol/L glyphosate concentration. Plant transformation was carried out with Dr. M.K. Reddy’s lab-modified protocol (unpublished). Regenerated seedlings from the transformations were transplanted into earthen pots covered with perforated polybags for hardening (at least for 3–5 days) inside the greenhouse conditions (ICGEB, New Delhi). All the positive plants, along with the control MTU1010 plants, were grown on soil under greenhouse conditions at 26 °C with a 14 h light/10 h dark cycle and a relative humidity of 60–70%. All the necessary fertilizers were applied to nourish the plants routinely and regularly irrigated to ensure proper growth. Healthy plant tissues were harvested and immediately frozen in liquid nitrogen prior to DNA and RNA isolation.

### 4.2. Preparation of the rcoSpCas9 Expression Cassette

A rice codon-optimized *Streptococcus pyogenes Cas9* (*rcoSpCas9*) was synthesized from GenScript (https://www.genscript.com). The sequence was initially cloned into Entry Clone 1 (-Amp, +Gen selection modified in Dr. MK Reddy’s Lab) with *Nco*I and *Not*I restriction sites. The *rcoSpCas9* was placed under the control of a constitutively expressing maize Ubiquitin promoter (ZmUbqP) (~1.99 kb, amplified using primers ZmUbq F/ZmUbq R) and a *Nopaline synthase* gene terminator (NosT) (~250 bp, amplified using primers nosT F/nosT R). We used *Kpn*I and *Nco*I restriction enzymes to clone the ZmUbq promoter and *Not*I and *Sac*I restriction enzymes for NosT, respectively. The resulting Entry vector 1 (EV1) harboring the *rcoSpCas9* expression cassette (ZmUbqP::rcoSpCas9:NosT) was then transferred into the pMDC99 binary vector, which harbored the *Hygromycin phosphotransferase* (*hpt*II) as a selection marker, using the gateway cloning method. A single round of LR recombination was employed to insert the full *rcoSpCas9* expression cassette (ZmUbiP:rcoSpCas9:NosT) between the left (LB) and right borders (RB) of the pMDC99 backbone (Figure S1). The final expression vector was transferred into the *Agrobacterium* strain (EHA105) by electroporation for rice plant transformation.

### 4.3. PCR Screening and Validation of rcoSpCas9 Overexpression Lines

To identify T0-generation positive *rcoSpCas9* overexpressed lines, 35 regenerated plants were used for PCR screening using two sets of primers: hygromycin (hptII F/hptII R) and the junction primer of the maize ubiquitin promoter and Cas9 CDS (Zm JN F/Cas9 JN R) (Table S1). To determine the copy number of transgene integration, stably transformed T1-generation seedlings were further analyzed using southern blotting, following a lab-modified protocol [24]. *EcoR*V restriction enzyme was used to digest the T-DNA segment for southern blot analysis. Souhtern-positive homozygous lines (E2, E10, and E20) were further used for western blot analysis using the Cas9 antibody. The Cas9 antibody was generated following a standard protocol at the ICGEB facility [48]. For antibody generation, we cloned *SpCas9* in the pET28a expression vector and expressed it in BL21 *E. coli* cells (Figure S2).

### 4.4. Designing and Cloning of Guide RNA and Repair Template

We used the CRISPRdirect (Yuki Naito, 2015) (https://crispr.dbcls.jp) online software for searching guide RNA target sites in the Indica rice genome, keeping all parameters as default. Two guide RNAs were designed to target the native copy of the rice *EPSP Synthase* gene for the substitution of Threonine to Isoleucine at position 173 (T173I) and Proline to Serine at position 177 (P177S) amino acids (together TIPS). These sites are present at the second exon, near the junction of the exon–intron site. Two 20 bp oligonucleotides (gTIPS F and gTIPS R, Table S1) sequences were selected at the genomic location of 1327 to 1345 bp of *EPSP synthase* gene from the position of start codon ATG. To drive the transcription of our synthesized guide RNAs (gTIPS), we used a rice-specific U6 promoter (OsU6_P) and a pol III terminator site at the end of the gRNA scaffold. To multiplex our guide RNA cloning system for future use, we synthesized the entire OsU6 promoter with an arbitrary sequence along with a guide RNA scaffold and pol III terminator from GeneScript under *Kpn*I and *Sac*I restriction sites. We incorporated 5′-CAGG-3′ in forward and 5′-AAAC-3′ at the reverse end of the arbitrary sequence to facilitate digestion with *BSa*I restriction enzyme and maintained similar overhangs for cloning 20 bp guide RNA, gTIPS. This synthesized construct was cloned into entry clone 1 (EV1, Gen^+^).

Further, we designed a repair template of 706 bp lengths with the substitution of T173I and P177S amino acids of the *EPSP synthase* gene. We also introduced several synonymous nucleotide substitutions without changing the amino acid residues at the target site to prevent Cas9 from re-targeting the donor fragment. Additionally, a unique *Hind*III restriction site was included in the repair template as a marker for validation in the edited lines.

### 4.5. Construction of Autonomously Replicating Deconstructed Wheat Dwarf Virus (dWDV)-Based Vector

We developed a binary expression cassette using a multi-round gateway cloning strategy to combine the expression cassette of OsU6 promoter::gRNA (gTIPS):: RNA pol III terminator with the repair template (repTIPS) under the control of autonomously replicating deconstructed wheat dwarf virus (dWDV) system for simultaneous delivery of guide RNA and repair template in the second transformation. The dWDV replicon system was designed with all the necessary components intact which are required for the autonomous viral replication i.e., small intergenic repeats (SIRs) and large intergenic repeats (LIRs). For the possibility of future multiplexing, we also introduced a multiple cloning site (MCS) consisting of restriction enzymes *Xho*I, *Nhe*I, *Xba*I, *Not*I, *Bgl*II, and *Sal*I sites. The system was first cloned into Entry clone 2 (EV2, Gen^+^, Chl^+^) under the *EcoR*I and *Sac*I restriction enzymes. This EV2-dWDV construct was later used for the cloning of our synthesized repair template (repTIPS), by replacing the WDV coat protein (CP) of dWDV using the *Xho*I and *Xba*II restriction sites. Later, multi-round gateway cloning was carried out to in vitro pyramiding the guide RNA construct and repair template into the modified pMDC99 (-*hpt*II) vector backbone.

### 4.6. Mutation Identification and Inheritance Analysis

Two sets of PCR primers (Table S1) were used for the amplification of the target region. The final PCR product or ~750 bp fragment was further used for the sequence analysis with the help of the Macrogen sequencing platform (https://www.macrogen.com). Seeds from the T0-edited lines were used for the mutation inheritance analysis via both germination assay (MS basal media with 50 µmol/L glyphosate) and foliar spray experiments with the herbicide Roundup (active ingredient: isopropylamine salt of glyphosate, 41.0% (*w*/*v*) with the doze of 2 mL/L). A foliar spray experiment was conducted at the initial tillering stage of the seedlings. The TIPS-edited plants along with the control WT MTU1010 plants were uniformly sprayed with commercial Roundup herbicide and maintained in a greenhouse at 26 °C controlled temperature, 14 h light/10 h dark cycles, and a relative humidity of 60–70%. The survivability was assessed one week after spraying. Control plants sprayed with water were used as a mock control.

### 4.7. Statistical Analysis

Statistical analyses were performed using one-way ANOVA, and the differences between means were compared using Tukey’s HSD test (*p* < 0.5) for the relevant datasets.

## 5. Conclusions

In summary, we successfully established a viral autonomous replicon system to deliver a donor template suitable for synchronizing double-strand DNA breaks through homology-based repair to achieve precise gene knock-in. In this study, we generated the TIPS mutation in the native *EPSP synthase* gene; these selective T173I and P177S substitutions disrupt glyphosate interaction with the EPSPS enzyme. The genetic engineering of herbicide-resistant rice plants provides a valuable approach to enable the use of environmentally safe herbicides with flexible application time during the entire crop-growing season, ensuring effective weed control. Moreover, the dWDV-based viral replicon system generated in this study demonstrates significant potential for delivering gene-editing components, thereby would be helpful to accelerate the process of precision breeding and advancing crop improvement strategies.

## Data Availability

All data are available in the research paper, along with the supplementary documents.

## Supplementary Materials

The following supporting information can be downloaded at: https://www.mdpi.com/article/10.3390/plants14030477/s1, Figure S1: Sequential steps for the generation of the rcoSpCas9 expression cassette; Figure S2: Cloning, expression, and purification of SpCas9 in *E. coli* BL21 cells; Figure S3: Generation of rcoCas9 stable lines under 50 mg/L Hygromycin selection, Table S1: The list of the primer pairs used in this research work.

## References

[1] Holme I.B., Gregersen P.L., Brinch-Pedersen H. (2019). Induced Genetic Variation in Crop Plants by Random or Targeted Mutagenesis: Convergence and Differences. Front. Plant Sci..

[2] Hasan M.M., Rafii M.Y., Ismail M.R., Mahmood M., Rahim H.A., Alam M.A., Ashkani S., Malek M.A., Latif M.A. (2015). Marker-Assisted Backcrossing: A Useful Method for Rice Improvement. Biotechnol. Biotechnol. Equip..

[3] Dunwell J.M. (2000). Transgenic Approaches to Crop Improvement. J. Exp. Bot..

[4] Bradford K.J., Van Deynze A., Gutterson N., Parrott W., Steven S. (2005). Regulating Transgenic Crops Sensibly: Lessons from Plant Breeding, Biotechnology and Genomics. Nat. Biotechnol..

[5] Mishra R., Joshi R.K., Zhao K. (2018). Genome Editing in Rice: Recent Advances, Challenges, and Future Implications. Front. Plant Sci..

[6] Mali P., Yang L., Esvelt K.M., Aach J., Guell M., DiCarlo J.E., Norville J.E., Church G.M. (2013). RNA-Guided Human Genome engineering via Cas9. Science.

[7] Cong L., Ran F.A., Cox D., Lin S., Barretto R., Habib N., Hsu P.D., Wu X., Jiang W., Marraffini L.A. (2013). Multiplex Genome Engineering Using CRISPR/Cas Systems. Science.

[8] Jinek M., Chylinski K., Fonfara I., Hauer M., Doudna J.A., Charpentier E. (2012). A Programmable Dual-RNA-Guided DNA Endonuclease in Adaptive Bacterial Immunity. Science.

[9] Lieber M.R. (2010). The Mechanism of Double-Strand DNA Break Repair by the Nonhomologous DNA End-Joining Pathway. Annu. Rev. Biochem..

[10] Stinson B.M., Loparo J.J. (2021). Repair of DNA Double-Strand Breaks by the Nonhomologous End Joining Pathway. Annu. Rev. Biochem..

[11] Dudáš A., Chovanec M. (2004). DNA Double-Strand Break Repair by Homologous Recombination. Mutat. Res. Rev. Mutat. Res..

[12] Yang H., Ren S., Yu S., Pan H., Li T., Ge S., Zhang J., Xia N. (2020). Methods Favoring Homology-Directed Repair Choice in Response to Crispr/Cas9 Induced-Double Strand Breaks. Int. J. Mol. Sci..

[13] Jung J.H., Seo Y.W. (2017). Challenges in Wide Implementation of Genome Editing for Crop Improvement. J. Crop Sci. Biotechnol..

[14] Denes C.E., Cole A.J., Aksoy Y.A., Li G., Neely G.G., Hesselson D. (2021). Approaches to Enhance Precise Crispr/Cas9-mediated Genome Editing. Int. J. Mol. Sci..

[15] Waterworth W.M., Drury G.E., Bray C.M., West C.E. (2011). Repairing Breaks in the Plant Genome: The Importance of Keeping It Together. New Phytol..

[16] Puchta H. (2005). The Repair of Double-Strand Breaks in Plants: Mechanisms and Consequences for Genome Evolution. J. Exp. Bot..

[17] Zhi-Qiang W., Feng X., Mu-Dan H.E., Hou-Peng W., Zhu Z.-Y., Yong-Hua S. (2015). Suppression of Ligase4 or XRCC6 Activities Enhances DNA Homologous Recombination Efficiency in Zebrafish Primordial Germ Cells. Acta Hydrobiol. Sin..

[18] Nishizawa-Yokoi A., Nonaka S., Saika H., Kwon Y.I., Osakabe K., Toki S. (2012). Suppression of Ku70/80 or Lig4 Leads to Decreased Stable Transformation and Enhanced Homologous Recombination in Rice. New Phytol..

[19] Nishizawa-Yokoi A., Cermak T., Hoshino T., Sugimoto K., Saika H., Mori A., Osakabe K., Hamada M., Katayose Y., Starker C. (2016). A Defect in DNA Ligase4 Enhances the Frequency of TALEN-Mediated Targeted Mutagenesis in Rice. Plant Physiol..

[20] Chen H., Neubauer M., Wang J.P. (2022). Enhancing HR Frequency for Precise Genome Editing in Plants. Front. Plant Sci..

[21] Mao Y., Botella J.R., Liu Y., Zhu J.K. (2019). Gene Editing in Plants: Progress and Challenges. Natl. Sci. Rev..

[22] Song F., Stieger K. (2017). Optimizing the DNA Donor Template for Homology-Directed Repair of Double-Strand Breaks. Mol. Ther. Nucleic Acids.

[23] Zhang J.P., Li X.L., Li G.H., Chen W., Arakaki C., Botimer G.D., Baylink D., Zhang L., Wen W., Fu Y.W. (2017). Efficient Precise Knockin with a Double Cut HDR Donor after CRISPR/Cas9-Mediated Double-Stranded DNA Cleavage. Genome Biol..

[24] Achary V.M.M., Sheri V., Manna M., Panditi V., Borphukan B., Ram B., Agarwal A., Fartyal D., Teotia D., Masakapalli S.K. (2020). Overexpression of Improved EPSPS Gene Results in Field Level Glyphosate Tolerance and Higher Grain Yield in Rice. Plant Biotechnol. J..

[25] Mikami M., Toki S., Endo M. (2015). Parameters Affecting Frequency of CRISPR/Cas9 Mediated Targeted Mutagenesis in Rice. Plant Cell Rep..

[26] Wada N., Ueta R., Osakabe Y., Osakabe K. (2020). Precision Genome Editing in Plants: State-of-the-Art in CRISPR/Cas9-Based Genome Engineering. BMC Plant Biol..

[27] Wang M., Lu Y., Botella J.R., Mao Y., Hua K., Zhu J.-k. (2017). Gene Targeting by Homology-Directed Repair in Rice Using a Geminivirus-Based CRISPR/Cas9 System. Mol. Plant.

[28] Čermák T., Baltes N.J., Čegan R., Zhang Y., Voytas D.F. (2015). High-Frequency, Precise Modification of the Tomato Genome. Genome Biol..

[29] Butler N.M., Baltes N.J., Voytas D.F., Douches D.S. (2016). Geminivirus-Mediated Genome Editing in Potato (*Solanum tuberosum* L.) Using Sequence-Specific Nucleases. Front. Plant Sci..

[30] Baltes N.J., Gil-Humanes J., Cermak T., Atkins P.A., Voytas D.F. (2014). DNA Replicons for Plant Genome Engineering. Plant Cell.

[31] Gelvin S.B. (2017). Integration of Agrobacterium T-DNA into the Plant Genome. Annu. Rev. Ofgenetics.

[32] Duke S.O., Powles S.B. (2009). Glyphosate-Resistant Crops and Weeds: Now and in the Future. AgBioForum.

[33] Schönbrunn E., Eschenburg S., Shuttleworth W.A., Schloss J.V., Amrhein N., Evans J.N.S., Kabsch W. (2001). Interaction of the Herbicide Glyphosate with Its Target Enzyme 5-Enolpyruvylshikimate 3-Phosphate Synthase in Atomic Detail. Proc. Natl. Acad. Sci. USA.

[34] Dong Y., Ng E., Lu J., Fenwick T., Tao Y., Bertain S., Sandoval M., Bermudez E., Hou Z., Patten P. (2019). Desensitizing Plant EPSP Synthase to Glyphosate: Optimized Global Sequence Context Accommodates a Glycine-to-Alanine Change in the Active Site. J. Biol. Chem..

[35] Yang S.H., Kim E., Park H., Koo Y. (2022). Selection of the High Efficient SgRNA for CRISPR-Cas9 to Edit Herbicide Related Genes, PDS, ALS, and EPSPS in Tomato. Appl. Biol. Chem..

[36] Jiang Y., Chai Y., Qiao D., Wang J., Xin C., Sun W., Cao Z., Zhang Y., Zhou Y., Wang X.C. (2022). Optimized Prime Editing Efficiently Generates Glyphosate-Resistant Rice Plants Carrying Homozygous TAP-IVS Mutation in EPSPS. Mol. Plant.

[37] Hummel A.W., Chauhan R.D., Cermak T., Mutka A.M., Vijayaraghavan A., Boyher A., Starker C.G., Bart R., Voytas D.F., Taylor N.J. (2018). Allele Exchange at the EPSPS Locus Confers Glyphosate Tolerance in Cassava. Plant Biotechnol. J..

[38] Rozas P., Kessi-Pérez E.I., Martínez C. (2022). Genetically Modified Organisms: Adapting Regulatory Frameworks for Evolving Genome Editing Technologies. Biol. Res..

[39] Hong J., Shi Q., Biswas S., Jiang S.C., Shi J. (2021). Moving Genome Edited Crops Forward from the Laboratory Bench to the Kitchen Table. Food Control.

[40] Niazi S.K. (2023). Gene Editing: The Regulatory Perspective. Encyclopedia.

[41] Calyxt Inc. (2016). Calyxt Completes Production of 30 Tons of Its High Oleic Soybean Product in Argentina.

[42] Menz J., Modrzejewski D., Hartung F., Wilhelm R., Sprink T. (2020). Genome Edited Crops Touch the Market: A View on the Global Development and Regulatory Environment. Front. Plant Sci..

[43] Zhang H., Zhang J., Wei P., Zhang B., Gou F., Feng Z., Mao Y., Yang L., Zhang H., Xu N. (2014). The CRISPR/Cas9 System Produces Specific and Homozygous Targeted Gene Editing in Rice in One Generation. Plant Biotechnol. J..

[44] Svitashev S., Young J.K., Schwartz C., Gao H., Falco S.C., Cigan A.M. (2015). Targeted Mutagenesis, Precise Gene Editing, and Site-Specific Gene Insertion in Maize Using Cas9 and Guide RNA. Plant Physiol..

[45] Peng A., Chen S., Lei T., Xu L., He Y., Wu L., Yao L., Zou X. (2017). Engineering Canker-Resistant Plants through CRISPR/Cas9-Targeted Editing of the Susceptibility Gene CsLOB1 Promoter in Citrus. Plant Biotechnol. J..

[46] Wang X., Tu M., Wang D., Liu J., Li Y., Li Z., Wang Y., Wang X. (2018). CRISPR/Cas9-Mediated Efficient Targeted Mutagenesis in Grape in the First Generation. Plant Biotechnol. J..

[47] Zhang Y., Massel K., Godwin I.D., Gao C. (2019). Applications and Potential of Genome Editing in Crop Improvement. Genome Biol..

[48] Harlow E.L.D. (1999). Using Antibodies: A Laboratory Manual.

