# Supplementary material for: A Gemini Virus-Derived Autonomously Replicating System for HDR-Mediated Genome Editing of the EPSP Synthase Gene in Indica Rice
Source: Plants (Basel). 2025 Feb 6;14(3):477. doi: 10.3390/plants14030477 (PMC11821261; doi:10.3390/plants14030477)
Supplement: Supplementary file 1 [file plants-14-00477-s001.zip › plants-3348866-supplementary.pdf]

plants

Article

A Gemini Virus-Derived Autonomously Replicating System for HDR-Mediated Genome Editing of the *EPSP Synthase* Gene in Indica Rice

Bhabesh Borphukan [1,2,*,†], Muslima Khatun [3,†], Dhirendra Fartyal [4], Donald James [5] and Malireddy K. Reddy [2]

1 Department of Crop and Soil Sciences, Washington State University, Pullman, WA 99163, USA
2 Crop Improvement Group, International Centre for Genetic Engineering and Biotechnology, New Delhi 110067, India
3 Molecular Biotechnology Division, National Institute of Biotechnology, Dhaka 1349, Bangladesh
4 Plant Nutrition Division, INRES, University of Bonn, 53113 Bonn, Germany
5 Department of Biotechnology, Forest Genetics and Biotechnology Division, Kerala Forest Research Institute, Thrissur 680653, India
* Correspondence: bhabesh.borphukan@wsu.edu
† These authors contributed equally to this work.

Figure S1

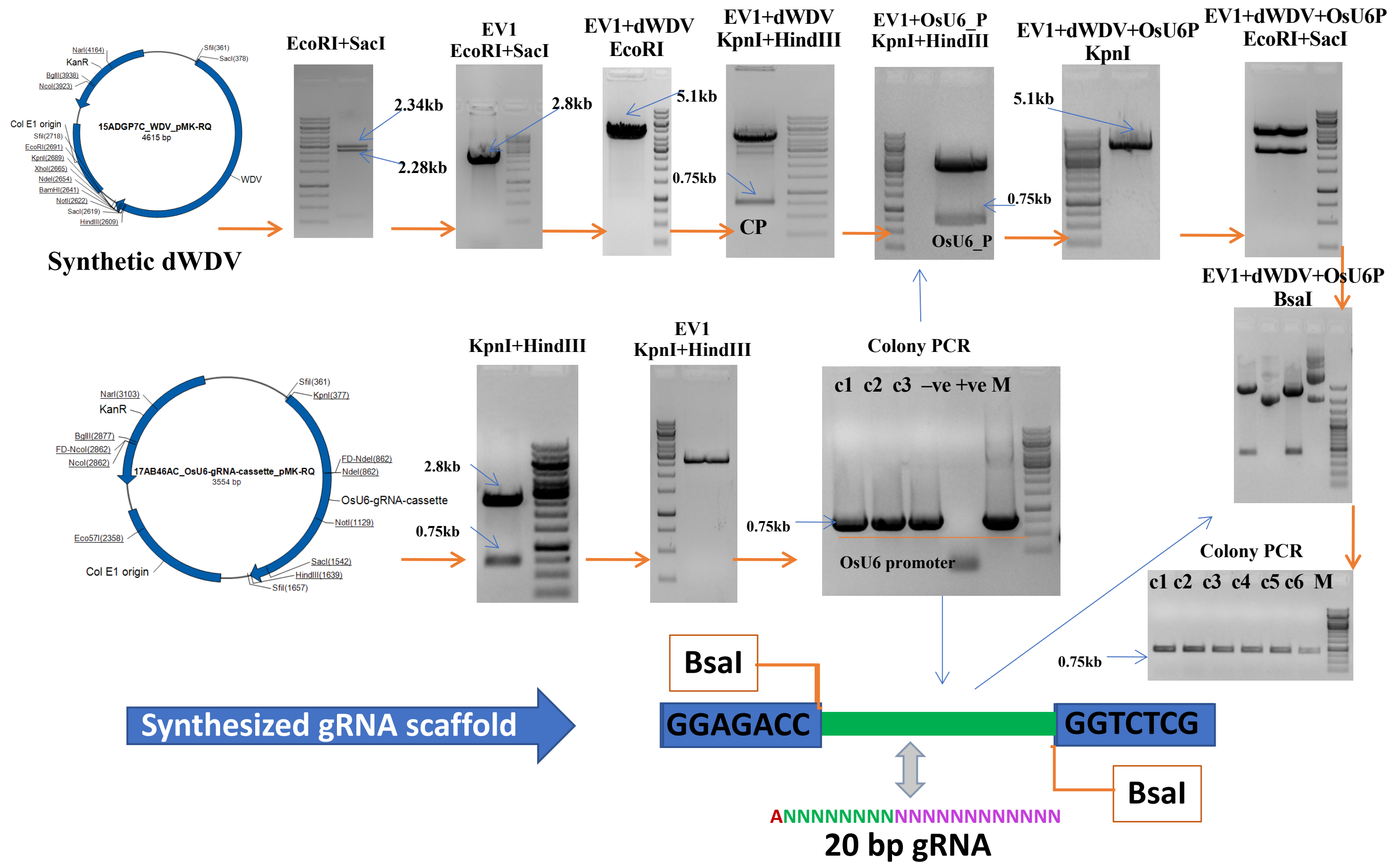

Figure S1: Sequential steps for the generation of the rcoSpCas9 expression cassette. This figure illustrates the cloning steps involved in constructing the rcoSpCas9 expression cassette using synthetic viral vectors. The top left shows the initial synthetic dWDV plasmid, which undergoes sequential restriction enzyme digestion and ligation, including steps with EcoRI + SacI, KpnI + HindIII, and BsaI, to introduce different elements of the expression cassette. Each step is verified by gel electrophoresis to confirm fragment sizes: 2.34 kb (dWDV), 2.88 kb (Linearized EV1), 5.1 kb (linearized EV1 after cloning dWDV fragment), and 0.75 kb (OsU6 promoter fragment digestion check after cloning into EV1) , as indicated.

In the lower section, another plasmid containing the OsU6-gRNA cassette is shown, constructed by cloning the gRNA scaffold and the 20 bp gRNA sequence with restriction digestion and ligation. Colony PCR (OsU6_F/OsU6_R) images verify successful incorporation of the cassette, with expected bands at 0.75 kb in positive clones. The synthesized gRNA scaffold is shown alongside the final assembly verification using BsaI digestion. GGAGACC and GGTCTCG are the overhanging sequences generated after BsaI digestion, which are also incorporated into the guide RNA primer sequences to enable seamless ligation. Primers used are provided in the supplementary table 1.

Figure S2

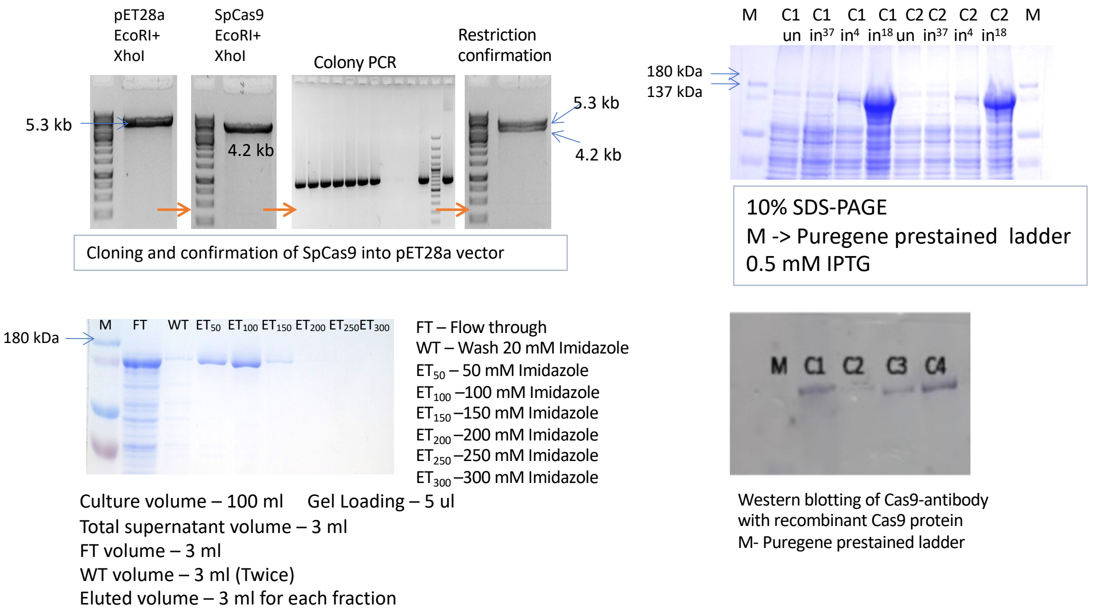

Figure S2: Cloning, expression, and purification of SpCas9 in E. coli BL21 cells.

This figure shows the cloning of SpCas9 (SpCas9 F/ SpCas9 R) into the pET28a vector using EcoRI and XhoI digestion, confirmed by gel electrophoresis with expected bands at 5.3 kb (vector backbone) and 4.2 kb (insert). Colony PCR (SpCas9 3′ F/ SpCas9 3′ R) and restriction digest confirm successful insertion.

For expression, E. coli BL21 cells with the SpCas9-pET28a construct were induced with 0.5 mM IPTG under different conditions (e.g., 37°C, 18°C), with SDS-PAGE revealing SpCas9 at ~180 kDa in induced samples.

Purification involved imidazole gradient elution (50–300 mM), with SDS-PAGE confirming the presence of SpCas9 in various elution fractions. Western blot analysis using Cas9-specific antibodies validated the presence of Cas9 in selected samples (C1–C4), with Puregene prestained ladder as a molecular weight reference. Primers used are provided in the supplementary table 1.

Figure S3

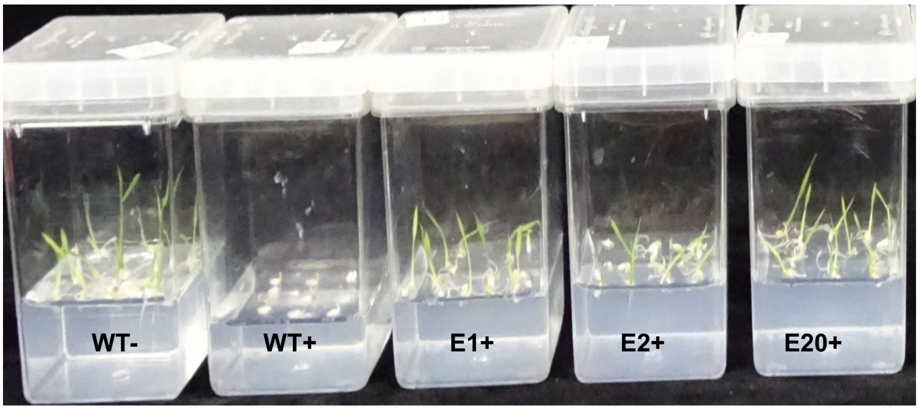

Figure S3: Generation of rcoCas9 stable lines under 50 mg/L Hygromycin selection
WT- MTU1010 control plants without Hygromycin.
WT+ MTU1010 control plants with 50 mg/L Hygromycin.
E1+/E2+/E20+ T0-seeds grown under 50 mg/L Hygromycin selection.

Table S1: The list of the primer pairs used in this research work

Sl. no	Primer name	Sequence (5'-3')
1	ZmUbq F	GGTACCCTGCAGTGCAGCGTGACCCG
2	ZmUbq R	CCATGGATCCAAAAGAAACAGTACCAAGC
3	SpCas9 F	CCATGGACTACAAGGACCACGACGG
4	SpCas9 R	TCACTTCTTTTTCTTGGCCTGGCCGG
5	nosT F	TAGAGCTCCGATCGTTCAAACATTTGGC
6	nosT R	CTTAAGCTTCCCGATCTAGTAACATAGAT
7	Zm JN F	AGAGATGCTTTTTGTTCGCTT
8	Cas9 JN R	GGTGTATCTGCGTCTGGCTGT
9	OsU6 F	TCATTAGCGGTATGCATGTT
10	OsU6 R	CATGGACATACCGCTTTA
11	OsEPSPS mut 1 F	GCACAACAGTGGTGGACAAC
12	OsEPSPS mut 1 R	TCCCTTGACACGAACAGGTG
13	OsEPSPS mut 2 F	ACGAGTCTGGTGAGGTCTGT
14	OsEPSPS mut 2 R	CGATCTCCACATCCCCAAGG
15	P1	GGTACCTGAGACTTTTCAACAAAGGG
16	P2	CCATGGTCAGCGTGTCCTCTCCAAAT
17	P3	AAGGATCCATGGCCTCTTCATCTGCAC
18	P4	ACGCGGCCGCACCCTCACACAATGACATCG
19	SpCas9 3′ F	CTCTCATCGAGACAAACGGC
20	SpCas9 3′R	GGAGCCCTTGAGCTTCTCGT
21	hptII F	ATGAAAAAGCCTGAACTCACC
22	hptII R	CTATTTCTTTGCCCTCGGAC
